# Adult onset multisystem Langerhans cell histiocytosis initially presenting with arginine vasopressin deficiency: a case report

**DOI:** 10.3389/fmed.2025.1643331

**Published:** 2025-12-01

**Authors:** Dan Zeng, Mingyu Hao, Liming Zhou, Yuli Wang, Junying Liu, Shujuan Chen, Litao Zheng, Yeyu Yang, Haiyan Li

**Affiliations:** 1 Department of Endocrinology, Shenzhen Scond People’s Hospital, The First Affiliated Hospital of Shenzhen University, Shenzhen Clinical Research Center for Metabolic Diseases, Shenzhen, China; 2 Department of Radiology, Shenzhen Second People's Hospital, Health Science Center of Shenzhen University, Shenzhen, China; 3 Guangzhou University of Chinese Medicine-Shenzhen Hospital Zhulin Community Health Service Station, Shenzhen, China

**Keywords:** Langerhans cell histiocytosis, pituitary stalk thickening, arginine vasopressin deficiency, multisystemic involvement, case report

## Abstract

**Background:**

Langerhans cell histiocytosis (LCH) is a rare hematopoietic disorder with diverse clinical presentations and sequential or concurrent multi-organ involvement. The adult onset is particularly uncommon and challenging to diagnose early.

**Case presentation:**

A 36-year-old female patient initially presented with polydipsia, polyuria, and dry mouth, gradually developing bone pain, facial erythema, and lymph node enlargement. Cranial MRI showed pituitary stalk thickening with the absence of a posterior pituitary signal. Iliac bone biopsy with immunohistochemistry demonstrated S-100(+), CD1a(+), and CD68(+) expression. After Systemic Chemotherapy and DDAVP administration, follow-up showed improvement of polyuria, polydipsia, and polyphagia, remission of osseous pain, regression of cutaneous manifestations, normalized pituitary stalk morphology, and reduced lesion metabolic activity.

**Conclusion:**

This report documents a complex and rare case to improve early diagnosis, reduce misdiagnosis, and prevent poor outcomes from delayed treatment.

## Introduction

Langerhans cell histiocytosis (LCH) represents a hematopoietic disorder characterized by the aberrant proliferation of immature dendritic cells, driven by the dysregulated activation of the mitogen-activated protein kinase (MAPK) signalling pathway (1, 2). The incidence is age-dependent, with pediatric rates of approximately 4.46 per million annually, while adult cases are significantly rarer at only 1.06 per million (3). Due to its insidious onset, diverse clinical manifestations, and the tendency for misdiagnosis resulting in poor prognosis, this article reports a case of adult-onset disease with arginine vasopressin deficiency (AVP-D) as the initial manifestation, ultimately diagnosed as LCH through bone lesion biopsy, providing a reference for early recognition and improved outcomes.

## Case presentation

In 2014, a 36-year-old obese female developed symptoms of polyuria, polydipsia, and polyphagia following diet and exercise-induced weight loss. Symptoms included hourly urination and menstrual irregularities. An initial pituitary MRI at another hospital revealed a pituitary lesion (approximately 0.5 cm × 0.5 cm × 0.3 cm) with a prolactin level of 13.04 μg/L (within the normal reference range of 3.34–26.72 μg/L). The patient underwent radiotherapy for presumptive prolactinoma (radiographic documentation and treatment protocol unavailable). Despite the intervention, clinical symptoms persisted post-treatment. Two weeks later, a Water Deprivation Test with DDAVP at a local hospital was positive, establishing a diagnosis of “AVP-D.” Electrolytes, parathyroid hormone, and growth hormone were normal upon testing. Oral desmopressin significantly improved her polyuria and polydipsia, with concurrent estrogen therapy for menstrual regulation.

**Figure 1:**
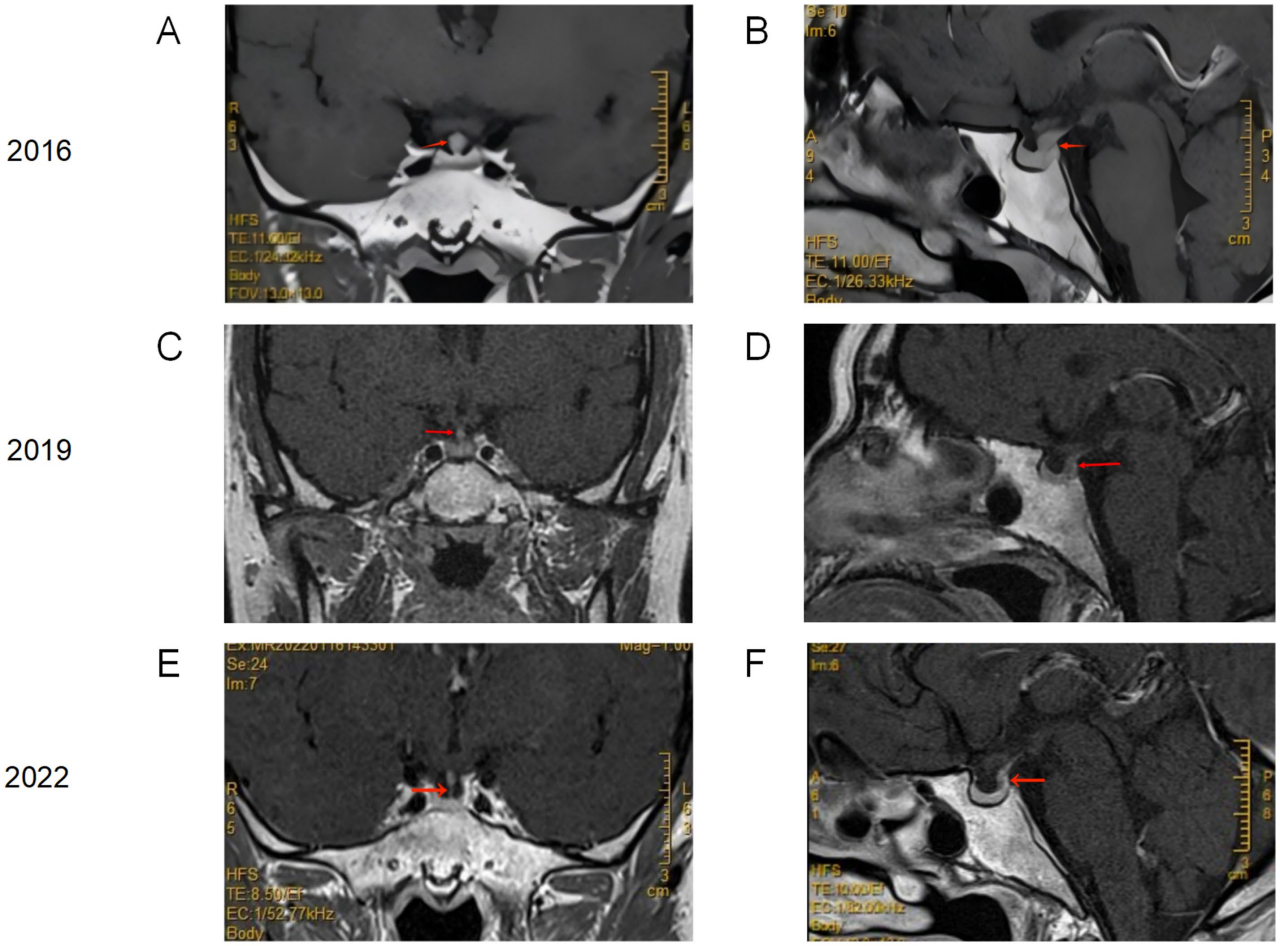
Longitudinal cranial MRI Follow-up of the LCH Patient. **(A,B)** Post-radiotherapy MRI of the pituitary gland (2016) demonstrating a slight reduction in pituitary volume with diffuse pituitary stalk thickening, uniform enhancement, and indistinct posterior pituitary visualization. **(C,D)** Underwent a follow-up cranial MRI at our hospital in 2019. Comparative analysis with imaging from February 16, 2016, demonstrates a subtle volumetric reduction of the pituitary gland. The infundibulum continues to exhibit nodular thickening with a homogeneous enhancement pattern, though notably diminished in diameter compared to the 2016 examination. **(E,F)** After being diagnosed with LCH and chemotherapy in 2022. Comparative analysis with imaging from December 17, 2019, reveals preserved adenohypophyseal dimensions. The pituitary infundibulum demonstrates physiological calibre with uniform morphology, homogeneous contrast enhancement, and absence of space-occupying lesions.

In 2016, the patient was hospitalized at a local medical center without other symptoms, where a repeat pituitary MRI revealed a lesion in the neurohypophyseal region (involving the pituitary stalk and infundibulum). During hospitalization at a local hospital, a follow-up pituitary MRI showed a slight reduction in pituitary volume with diffuse pituitary stalk thickening, uniform enhancement, and indistinct posterior pituitary visualization (Figure 1). The patient was diagnosed with complete AVP-D and post-radiation status. Subsequently, the patient continued on oral desmopressin therapy.

In 2017, she experienced left hip pain but did not undergo further examination or treatment. In November 2019, owing to progressively worsening bone pain and the development of a facial rash, she presented to our orthopaedic department. A pelvic MRI revealed pathological changes in the left hip bone, lymph nodes adjacent to the left iliac vessels, and inguinal lymph nodes. Bone lesion biopsy indicated eosinophilic granuloma of the left pelvis (Supplementary Figure 1).

No relevant past medical history or family history was reported.

Physical examination demonstrated bilateral symmetrical facial erythema, left hip deep tenderness, restricted range of motion in left knee flexion and extension, left lower limb shortening of approximately 2 cm relative to the right side, and significant left lower limb gait abnormality.

The patient was later admitted to our department on December 16, 2019, for further investigations (Table 1). Elevated ACTH and urinary free cortisol (24-h), with other laboratory tests, including blood cell counts, blood cell counts, cortisol, growth hormone, insulin-like growth factor-1 (IGF-1), parathyroid hormone (PTH), liver and kidney function, electrolytes, and bone metabolism markers were all within normal limits. Pituitary MRI showed: a slight reduction in pituitary volume compared to previous studies; the pituitary stalk still demonstrated nodular thickening with uniform enhancement, which had decreased in size compared to 2016 (Figure 1).

**Table 1:** Pituitary assessment, bone turnover markers and tumor markers.

Items	Result	Reference range
Pituitary assessment		
IGF-1	0.011	0.010–3.607
ACTH (8 a.m.)	69.20	0–46 pg./mL
Serum Cortisol (8 a.m.)	14.06	3.09–22.4 μg/dL
Urinary Free Cortisol(24-h)	403.00	28.5–213.7 μg/dL
PRL	5.65	3.34–26.72 ng/mL
FSH	6.62	Follicular phase: 3.85–8.78 IU/L; Ovulatory phase: 4.54–22.51 IU/L; Luteal phase: 1.79–5.12 IU/L
LH	7.20	Follicular phase: 2.12–10.89 IU/L; Ovulatory phase: 19.8–103 IU/L; Luteal phase: 1.2–12.86 IU/L
E2	35.00	Follicular phase: 23–139 pg./mL; Ovulatory phase: 83–495 pg./mL; Luteal phase: 42–338 pg./mL
Testosterone	0.17	0.1–0.75 ng/mL
Urine Specific Gravity	1.003	1.002–1.030
Urine Osmolality	205	400–1,000 mOsm/kg
Bone turnover markers		
25-(HO)D	25.72	11–48 ng/mL
P1NP	45.10	15.13–76.31 ng/mL
PTH	43.38	15–65 pg./mL
Collagen Specific Sequence	0.63	0.030–1.008 ng/mL
N-MID	19.03	11–48 ng/ml
Tumor markers		
AFP	1.9	0–8.1 ng/mL
CEA	<0.50	0–5 ng/mL
Ferritin	39.9	10–291 ng/mL
CA125	4.0	0–35 U/mL
CA199	3.93	0–37 U/mL
CA153	<0.5	0–38.6 U/mL
Blood cell counts		
WBC	7.96	10^9/L
RBC	3.97	10^12/L
HB	113.0	g/L
PLT	304.0	10^9/L

IGF-1, Insulin-like Growth Factor-1; ACTH, Adrenocorticotropic Hormone; PRL, Prolactin; FSH, Follicle-Stimulating Hormone; LH, Luteinizing Hormone; E2, Estradiol; 25-(HO)D, 25-Hydroxyvitamin D. P1NP, Total Collagen Type I Aminoterminal Propeptide. PTH, Parathyroid Hormone; N-IMD, N-terminal Mid-fragment; WBC, White Blood Cell; RBC, Red Blood Cell; HB, Hemoglobin; PLT, Platelet.

**Figure 2:**
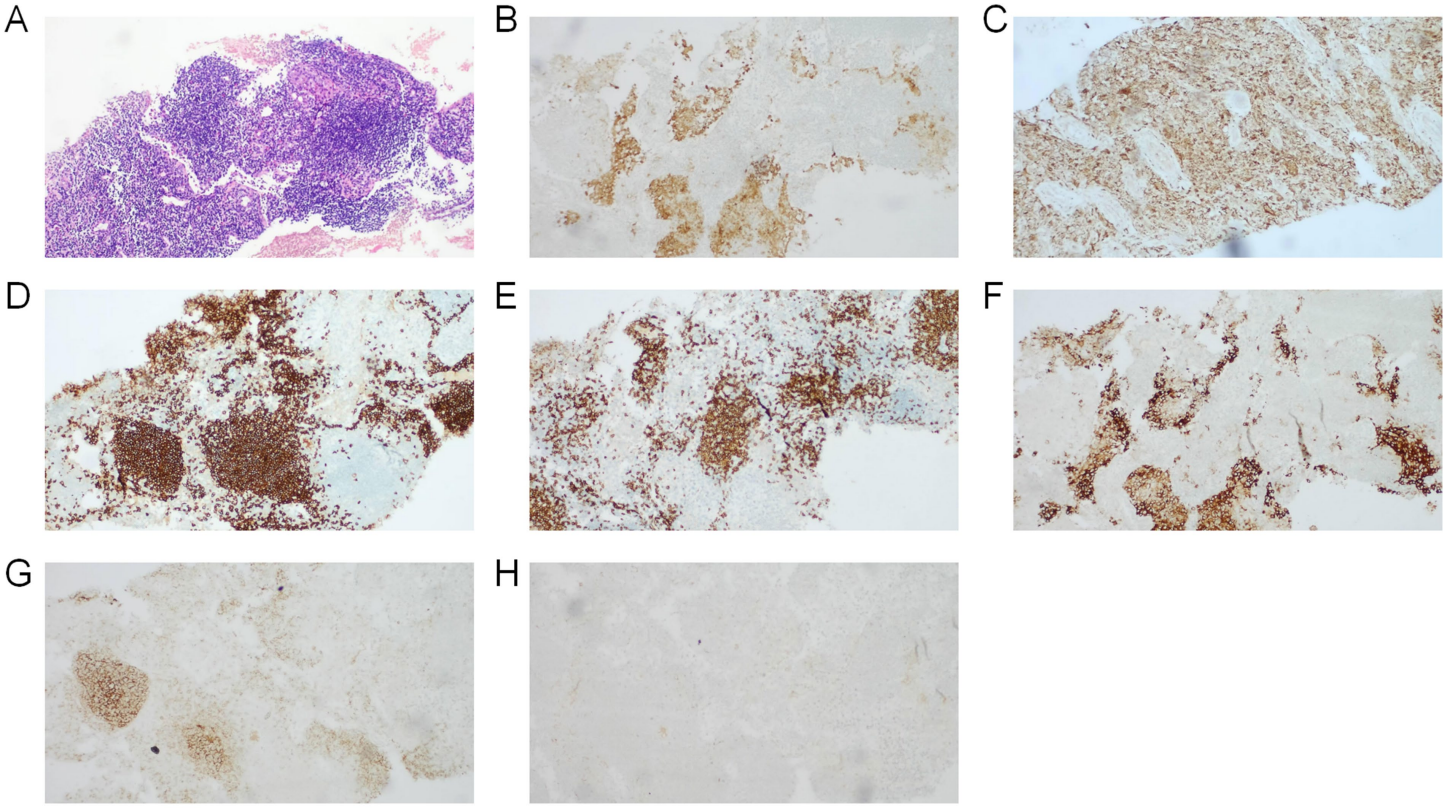
Images demonstrate the typical histology of an LCH lesion obtained from a bone biopsy. **(A)** Hematoxylin and eosin stains demonstrated fine chromatin with visible nucleoli, exhibiting diffuse or nodular distribution with prominent eosinophilic infiltration, accompanied by neutrophils, lymphocytes, and multinucleated macrophages. Immunohistochemistry positive for **(B)** S-100, **(C)** CD68, **(D)** CD2scattered, **(E)** CD3, **(F)** CD1a. Immunohistochemistry negative for **(G)** CD21, **(H)** CK.

Considering the patient’s pituitary stalk thickening and symptoms of polyuria, dry mouth, polydipsia, bone pain, facial erythema, and lymphadenopathy, tumor markers were further evaluated in addition to pituitary function assessment (Table 1). PET-CT (Supplementary Figure 2) revealed Langerhans cell histiocytosis involving multiple skeletal sites and lymph nodes throughout the body. Histopathological examination of the bone lesion biopsy (Figure 2) confirmed the diagnosis of Langerhans cell histiocytosis.

Based on the combined findings from MRI, PET-CT, and histopathological results, the definitive diagnosis was established as multisystem Langerhans cell histiocytosis (involving pituitary, osseous structures, lymph nodes, and cutaneous tissues). The patient received oral desmopressin 0.1 mg three times daily for symptom management and received regular chemotherapy from the hematology department for 1 year.

Follow-up in 2020: The patient showed significant improvement in left hip pain, normal menstrual cycle, resolution of facial erythema, and no obvious thirst, polydipsia, or polyuria. PET-CT (Supplementary Figure 2) demonstrated that multiple Langerhans cell histiocytosis lesions were in a suppressed state following treatment. Repeat cranial MRI (Figure 1) in 2022 showed stable pituitary volume with no interval changes; the pituitary stalk demonstrated normal thickness and homogeneous enhancement without mass lesions.

**Figure 3:**
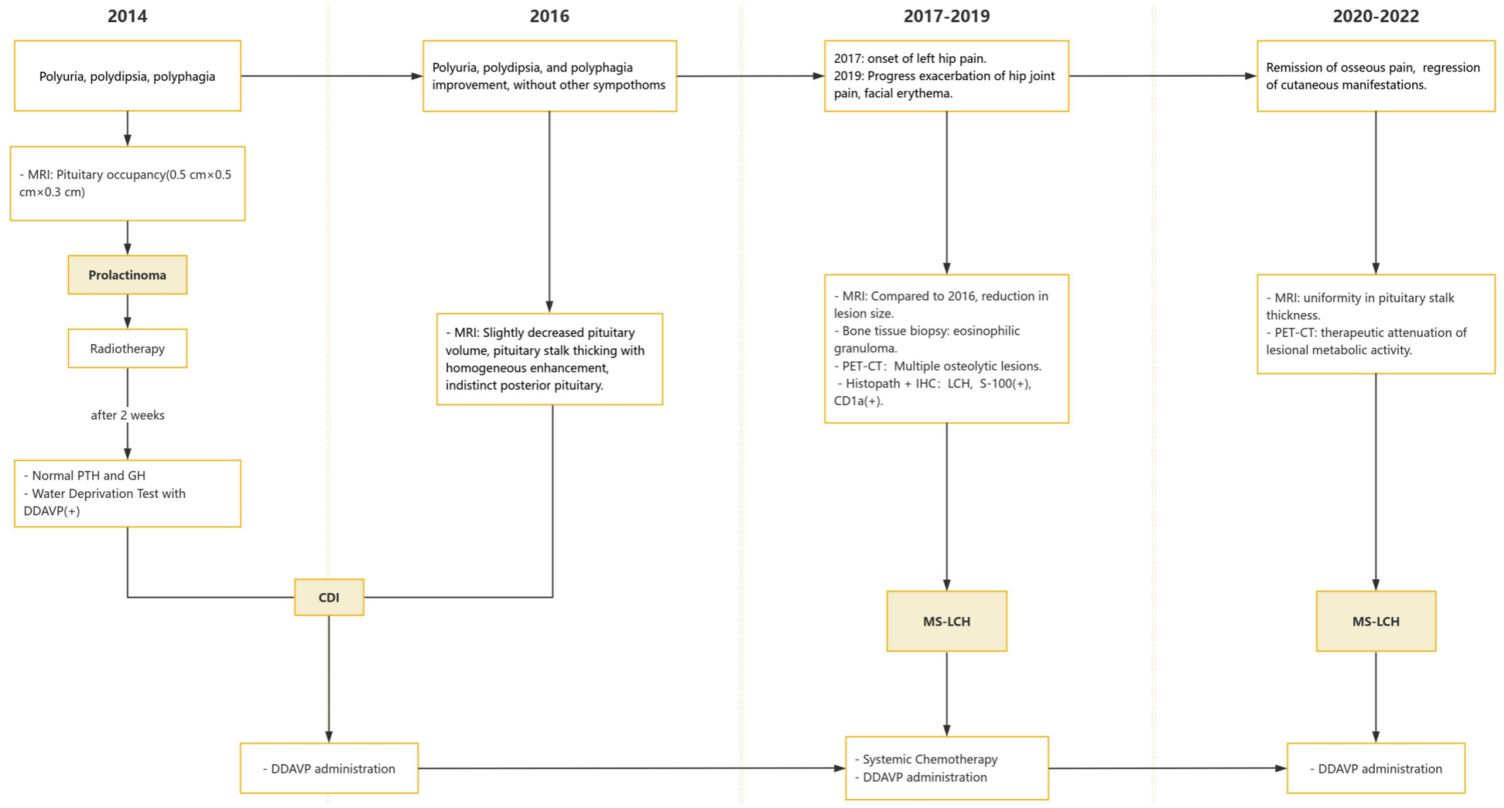
Appointment schedule. MS-LCH, multisystem LCH; PRL, Prolactin; AVP-D, Arginine vasopressin deficiency; DDAVP, Desmopressin acetate.

The whole timeline of clinical progression is shown in Figure 3. Pituitary assessment, bone turnover markers and tumor markers are shown in Table 1.

## Discussion

LCH is a rare neoplastic disorder of bone marrow origin whose etiology remains incompletely understood. It is characterized by abnormal proliferation and infiltration of Langerhans cells into multiple organs (1). The lesions can involve multiple systems either sequentially or simultaneously, with bone (approximately 80%), skin (approximately 33%), and pituitary (approximately 25%) being the most commonly affected sites (2). While involvement of lungs, thyroid, liver, spleen, female reproductive system, hematopoietic system, and lymph nodes is relatively rare, pulmonary involvement shows a markedly higher prevalence in adult patients compared to pediatric cases. The clinical presentations of LCH are complex and varied, rendering diagnosis difficult. Based on the 2021 international expert consensus (4), LCH is categorized into four subtypes: single-lesion LCH, pulmonary isolated LCH, single-system multifocal LCH, and multisystem LCH. Treatment strategies and first-line drugs differ significantly among different subtypes. When risk organs such as the hematopoietic system, liver, and spleen are involved in pediatric LCH patients, treatment response is poor and patient prognosis is generally unfavourable (5, 6), but remains undefined in adults. The classic cutaneous presentations of LCH include red/brown rashes, papules, or ulcers accompanied by scaling or crusting (7, 8). While LCH primarily affects children, recent research demonstrates that the disease may also manifest in adults, though with a relatively low incidence of merely 1.06 per 1,000,000 (3). Among adults, LCH is uncommon and presents with varied clinical features, with AVP-D typically being the first endocrine manifestation (9). The natural progression of adult LCH is not completely understood, but studies suggest that the disease may extend from the pituitary to more extensive areas of the central nervous system (10). As defined in the consensus by Goyal et al. (2), the diagnostic criteria for multisystem LCH are the involvement of ≥2 organs or organ systems. This patient presented with multisystem involvement, initially manifesting as pituitary lesions with xerostomia, polydipsia, polyuria, and pituitary stalk thickening; subsequently developed skeletal involvement characterized by multiple osteolytic lesions and bone pain; and later developed lymph node and skin lesions manifesting as multiple lymphadenopathies and facial erythema.

The diagnosis of LCH requires consideration of clinical manifestations, radiological features, and pathological examination. Classic immunological markers comprise positive cellular immunostaining for CD1a, CD207, and S100 (1, 2, 4, 11). For challenging diagnostic cases, advanced imaging techniques such as PET-CT can assist in determining the extent of lesions (12, 13). Regarding treatment, local therapy is recommended for LCH patients with single-lesion involvement. Systemic chemotherapy or radiotherapy is preferably recommended for multisystem LCH and multifocal bone involvement (14). Additionally, bisphosphonate treatment for multifocal bone involvement has been proven to effectively alleviate symptoms (15). Prognosis varies due to the complexity of the disease course, with single-system LCH patients showing favorable outcomes under appropriate treatment, while multisystem cases have a 5-year overall survival rate of approximately 90% (2), particularly Hepatic or hematological involvement may indicate poor prognosis (16). The patient’s diagnosis was established based on an iliac bone biopsy, with pathological results indicating Langerhans cell histiocytosis. Immunohistochemical results showed CD1a and S100 protein positivity. Following radiotherapy and regular chemotherapy, the patient’s left hip pain significantly improved, facial erythema resolved, and symptoms of polydipsia, excessive drinking, and polyuria disappeared; cranial MRI showed uniform thickness of the pituitary stalk. PET-CT indicated lesion suppression after treatment.

Since LCH involves multiple systems and clinical manifestations often resemble other diseases, diagnosis is challenging. Particularly in adult patients, LCH lacks clear clinical symptoms for extended periods, leading to relatively severe disease at the time of initial diagnosis. Timely management is crucial, as early identification and treatment can reduce endocrine dysfunction and other potential systemic damage, improving patients’ quality of life (1, 14). Cases that fail to receive timely treatment may develop serious complications, such as bone marrow or pulmonary infiltration, further affecting prognosis (17).

This report presents a case of an adult-onset patient with AVP-D as the initial symptom, accompanied by a pituitary mass lesion. Early differential diagnosis of the pituitary mass etiology was not performed in detail, nor was screening for other systemic involvement conducted. It was not until the patient developed obvious bone pain, facial erythema, and lymphadenopathy that further PET-CT revealed multiple osteolytic lesions and lymph node infiltration. The diagnosis of Langerhans cell histiocytosis was confirmed through pathological biopsy of the iliac bone lesion. Following diagnosis, the facial skin erythema and multiple lymphadenopathies were identified as manifestations of multisystem involvement of this disease. Through timely and regular chemotherapy, the condition was effectively controlled, improving the patient’s quality of life. Due to typical early symptoms and timely diagnosis, this patient received appropriate early treatment regimens, significantly improving prognosis. This study has limitations regarding incomplete clinical data during the early disease onset, but this did not significantly impact the final diagnosis and treatment planning. Additionally, the specific parameters and administration details of the chemotherapy regimen were not sufficiently detailed, with genetic analysis for BRAF not performed, limiting the comprehensive evaluation of treatment efficacy.

## Conclusion

In conclusion, LCH poses significant diagnostic and therapeutic challenges due to its unknown etiology and diverse clinical manifestations affecting multiple organ systems, with a rare adult-onset that is prone to misdiagnosis and missed diagnosis. Particularly in adults, the diagnostic and therapeutic challenges are enormous. Definitive diagnosis of LCH relies on pathological examination, combined with endocrine function assessment, imaging, and histopathological testing, providing the accurate foundation for early identification and promising to improve patient diagnostic and therapeutic outcomes and quality of life.

## Data Availability

The original contributions presented in the study are included in the article/Supplementary material, further inquiries can be directed to the corresponding author.

## Supplementary material

SUPPLEMENTARY FIGURE 1 Pelvic MRI in 2019 revealed abnormal osseous changes in the left hip **(A)**, suspicious for malignancy, and multiple enlarged lymph nodes in the para-iliac vascular and inguinal regions **(B)**.

SUPPLEMENTARY FIGURE 2 Pre- and post-treatment PET/CT Images. Upper row **(A–C)**: Baseline 18F-FDG PET/CT imaging (2019) acquired prior to chemotherapy initiation revealed mild hypermetabolic activity within multiple lymphadenopathy and osseous lesions of the left iliac bone, consistent with multifocal nodal involvement of Langerhans cell histiocytosis. **(A)** Coronal PET showing hypermetabolic lesion in the left hip region. **(B)** Axial CT demonstrating osteolytic destruction of the left acetabulum and pubic bone. **(C)** Fused PET/CT confirming abnormal glucose metabolism in the left acetabular and pubic region. Lower row **(D–F)**: Post-treatment PET/CT images (2020). Compared to pre-treatment imaging (December 2019), left iliac bone lesions showed no significant changes in size, morphology, or metabolic activity, with multiple residual foci. Lymph node lesions demonstrated reduced metabolic uptake and modest volume reduction, indicating partial suppression of multifocal Langerhans cell histiocytosis following therapy. **(D)** Coronal PET showing significantly decreased metabolic activity in the left hip lesion. **(E)** Axial CT revealing sclerotic margins and volume reduction of the lesion in the left acetabulum and pubic bone. **(F)** Fused PET/CT demonstrating markedly reduced metabolic activity, consistent with post-treatment suppression.

## References

[1] Carlos Rodriguez-Galindo C Allen CE. Langerhans cell histiocytosis. Blood. (2020) 135:1319–31. doi: 10.1182/blood.2019000934, PMID: 32106306

[2] Goyal G Tazi A Go RS Rech KL Picarsic JL Vassallo R . International expert consensus recommendations for the diagnosis and treatment of Langerhans cell histiocytosis in adults. Blood. (2022) 139:2601–21. doi: 10.1182/blood.2021014343, PMID: 35271698 PMC11022927

[3] Liu H Stiller CA Crooks CJ Rous B Bythell M Broggio J . Incidence, prevalence and survival in patients with Langerhans cell histiocytosis: a national registry study from England, 2013–2019. Br J Haematol. (2022) 199:728–38. doi: 10.1111/bjh.18459, PMID: 36122574 PMC9826274

[4] Gulati N Allen CE. Langerhans cell histiocytosis: version 2021. Hematol Oncol. (2021) 39:15–23. doi: 10.1002/hon.2857, PMID: 34105821 PMC9150752

[5] Tillotson C.V. Reynolds S.B. Patel B.C., Langerhans cell histiocytosis. (2025). In: StatPearls. Treasure Island (FL): StatPearls Publishing. 28613635

[6] Sato A Kobayashi M Yusa N Ogawa M Shimizu E Kawamata T . Clinical and prognostic features of Langerhans cell histiocytosis in adults. Cancer Sci. (2023) 114:3687–97. doi: 10.1111/cas.15879, PMID: 37364599 PMC10475785

[7] Iraji F Poostiyan N Dehnavi PR Soghrati M. Langerhans cell histiocytosis: a case report with unusual cutaneous manifestation. Adv Biomed Res. (2018) 7:102. doi: 10.4103/abr.abr_119_17 30050890 PMC6036769

[8] Lian C Lu Y Shen S. Langerhans cell histiocytosis in adults: a case report and review of the literature. Oncotarget. (2016) 7:18678–83. doi: 10.18632/oncotarget.7892, PMID: 26942568 PMC4951319

[9] Moszczyńska E Kunecka K Baszyńska-Wilk M Perek-Polnik M Majak D Grajkowska `W. Pituitary stalk thickening: causes and consequences. The children’s memorial health institute experience and literature review. Front Endocrinol. (2022) 13:13. doi: 10.3389/fendo.2022.868558, PMID: 35669693 PMC9163297

[10] Kadowaki Y Nishiyama M Nakamura M Morisaka H Fujimoto S Terada Y . Adult-onset Langerhans cell histiocytosis changing CNS lesion from pituitary to suprasellar extension. Endocrinol Diabetes Metab Case Rep. (2022) 2022:EDM22-0232. doi: 10.1530/EDM-22-0232, PMID: 35642690 PMC9175615

[11] Cerbone M Visser J Bulwer C Ederies A Vallabhaneni K Ball S . Management of children and young people with idiopathic pituitary stalk thickening, central diabetes insipidus, or both: a national clinical practice consensus guideline. Lancet Child Adolesc Health. (2021) 5:662–76. doi: 10.1016/S2352-4642(21)00088-2, PMID: 34214482

[12] Devuyst F Kazakou P Balériaux D Alexopoulou O Burniat A Salenave S . Central diabetes insipidus and pituitary stalk thickening in adults: distinction of neoplastic from non-neoplastic lesions. Eur J Endocrinol. (2020) 183:95–105. doi: 10.1530/EJE-20-0058 32530258

[13] Baratto L Nyalakonda R Theruvath AJ Sarrami AH Hawk KE Rashidi A . Comparison of whole-body DW-MRI with 2-[18F]FDG PET for staging and treatment monitoring of children with Langerhans cell histiocytosis. Eur J Nucl Med Mol Imaging. (2023) 50:1689–98. doi: 10.1007/s00259-023-06122-6, PMID: 36717409 PMC10121877

[14] Kobayashi M Tojo A. Langerhans cell histiocytosis in adults: advances in pathophysiology and treatment. Cancer Sci. (2018) 109:3707–13. doi: 10.1111/cas.13817, PMID: 30281871 PMC6272080

[15] Georgakopoulou D Anastasilakis AD Makras P. Adult Langerhans cell histiocytosis and the skeleton. J Clin Med. (2022) 11:909. doi: 10.3390/jcm11040909, PMID: 35207181 PMC8875624

[16] Lu Y Liu L Wang Q Liu B Zhao P Guan G . Clinical features and prognostic factors of pediatric Langerhans cell histiocytosis: a single-center retrospective study. Front Med (Lausanne). (2024) 11:1452003. doi: 10.3389/fmed.2024.1452003 39882514 PMC11774849

[17] Goyal G Acosta-Medina AA Abeykoon JP Dai C Ravindran A Vassallo R . Long-term outcomes among adults with Langerhans cell histiocytosis. Blood Adv. (2023) 7:6568–78. doi: 10.1182/bloodadvances.2023010706, PMID: 37698994 PMC10641096

