# Supplementary figures and images for: Adult onset multisystem Langerhans cell histiocytosis initially presenting with arginine vasopressin deficiency: a case report
Source: Front Med (Lausanne). 2025 Dec 1;12:1643331. doi: 10.3389/fmed.2025.1643331 (PMC12702692; doi:10.3389/fmed.2025.1643331)

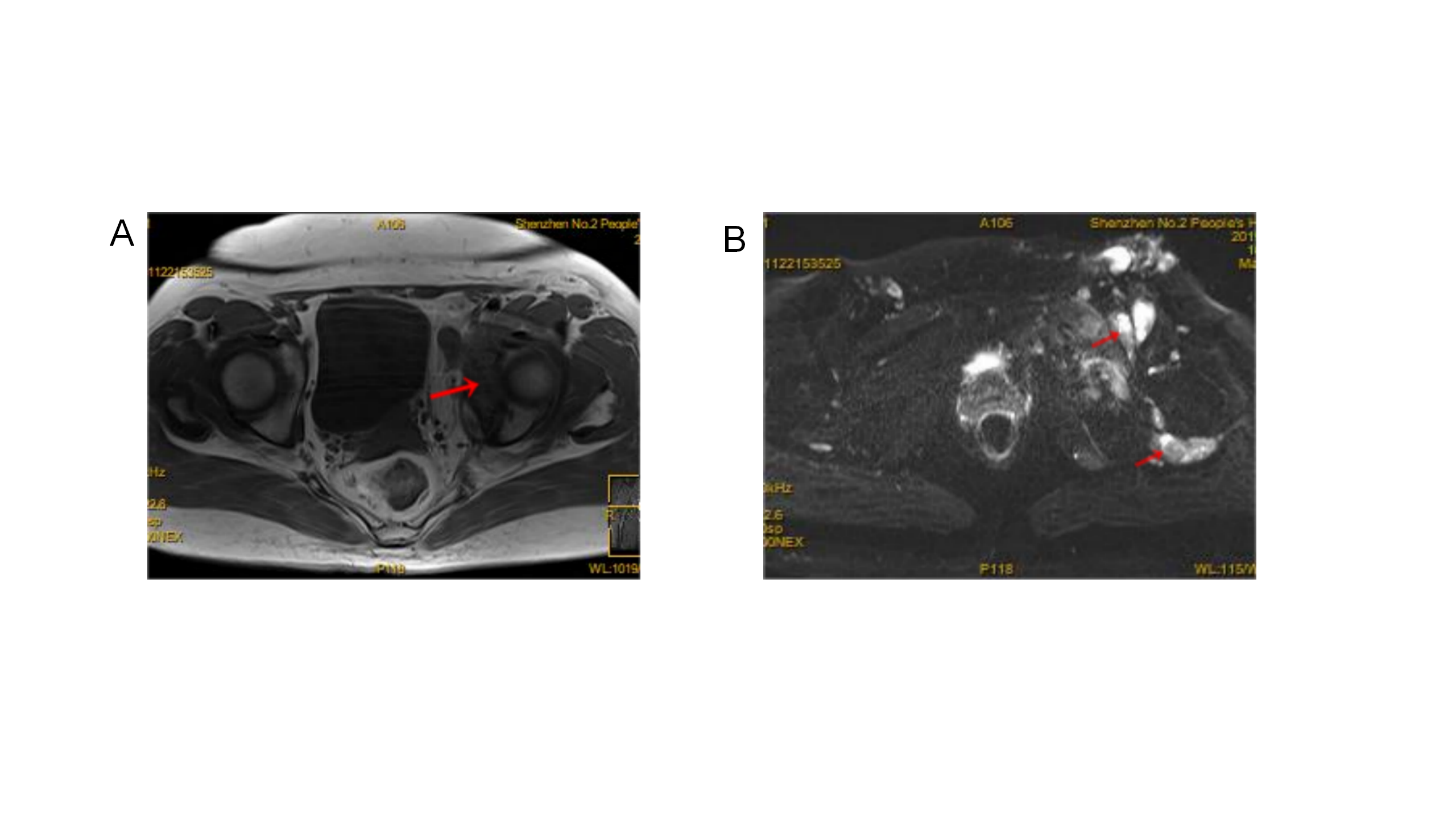

Supplement: SUPPLEMENTARY FIGURE 1 — Pelvic MRI in 2019 revealed abnormal osseous changes in the left hip (A), suspicious for malignancy, and multiple enlarged lymph nodes in the para-iliac vascular and inguinal regions (B). [file Image_1.TIF]

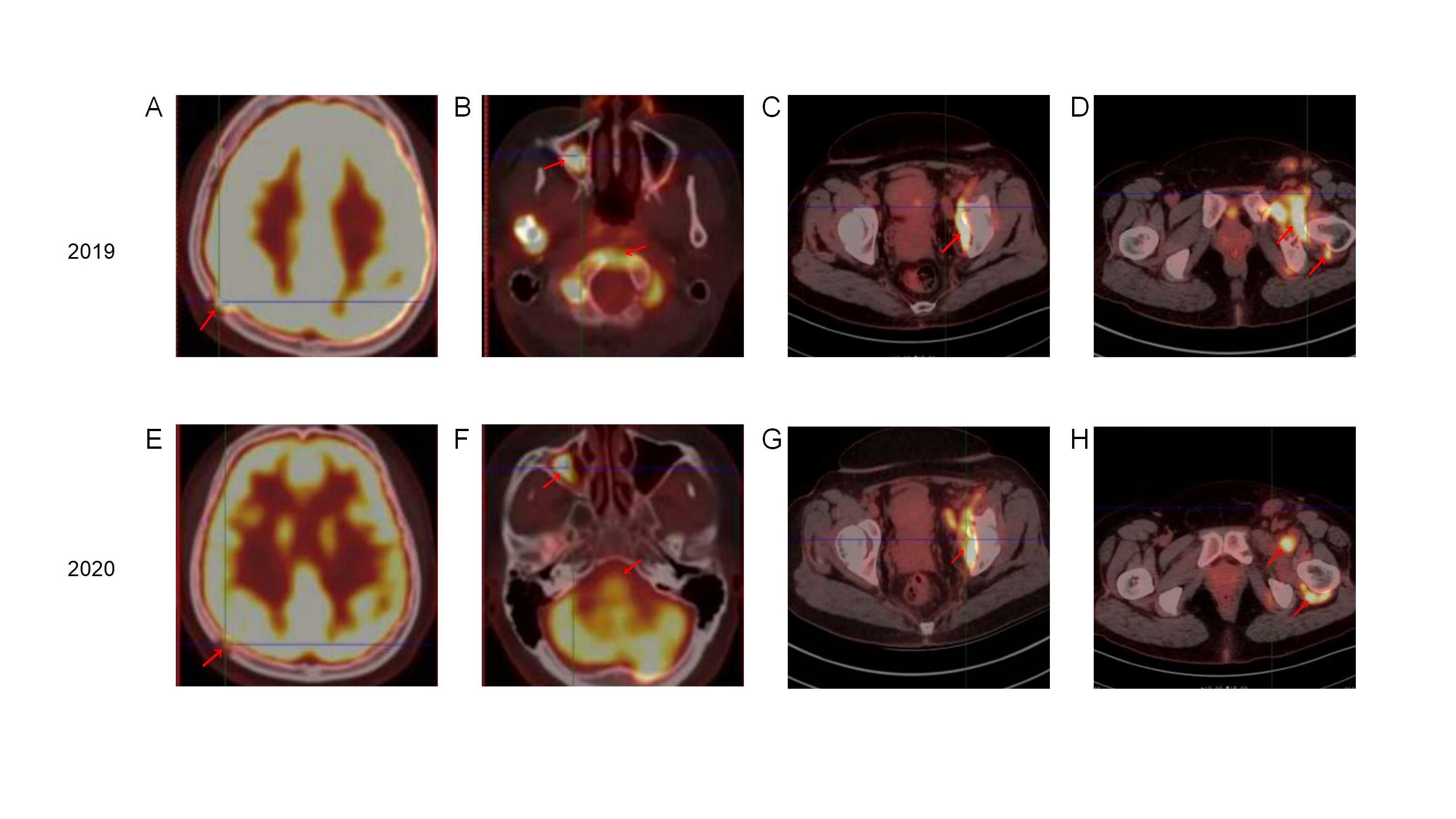

Supplement: SUPPLEMENTARY FIGURE 2 — Pre- and post-treatment PET/CT Images. Upper row (A–C): Baseline 18F-FDG PET/CT imaging (2019) acquired prior to chemotherapy initiation revealed mild hypermetabolic activity within multiple lymphadenopathy and osseous lesions of the left iliac bone, consistent with multifocal nodal involvement of Langerhans cell histiocytosis. (A) Coronal PET showing hypermetabolic lesion in the left hip region. (B) Axial CT demonstrating osteolytic destruction of the left acetabulum and pubic bone. (C) Fused PET/CT confirming abnormal glucose metabolism in the left acetabular and pubic region. Lower row (D–F): Post-treatment PET/CT images (2020). Compared to pre-treatment imaging (December 2019), left iliac bone lesions showed no significant changes in size, morphology, or metabolic activity, with multiple residual foci. Lymph node lesions demonstrated reduced metabolic uptake and modest volume reduction, indicating partial suppression of multifocal Langerhans cell histiocytosis following therapy. (D) Coronal PET showing significantly decreased metabolic activity in the left hip lesion. (E) Axial CT revealing sclerotic margins and volume reduction of the lesion in the left acetabulum and pubic bone. (F) Fused PET/CT demonstrating markedly reduced metabolic activity, consistent with post-treatment suppression. [file Image_2.TIF]
